# Correlation of miR-31 and miR-373 expression with KRAS mutations and its impact on prognosis in colorectal cancer

**DOI:** 10.1186/s43046-022-00136-1

**Published:** 2022-08-22

**Authors:** Hasan Ashoori, Shaghayegh Kamian, Farnaz Vahidian, Mohammad Ebrahim Ghamarchehreh

**Affiliations:** 1 Human Genetics Research Center, Baqiyatallah University of Medical Sciences, Tehran, Iran; 2 Department of Radiation Oncology, School of Medicine, Shahid Beheshti University of Medical Sciences, Tehran, Iran; 3 Department of Biology, Science and Arts University, Yazd, Iran; 4 Baqiyatallah Research Center of Gastroentrology and Liver Disease, Baqiyatallah University of Medical Sciences, Tehran, Iran

**Keywords:** Colorectal neoplasms, Genes, RAS, Proto-oncogene proteins p21(RAS), MIRN31 microRNA, human, MIRN373 microRNA, human

## Abstract

**Introduction:**

Colorectal cancers (CRC) are among the most common cancers. There are different modalities for treatment including chemotherapy, surgery, and radiotherapy. There are some mutations in cancers which can assist in the treatment and better prognosis of patients. In this study, two molecular markers (miR-31 and miR-373) were involved in the pathogenesis of CRC and their association with histopathological features was investigated. As well, the prognostic value of these molecular markers was investigated in CRC patients with or without common KRAS mutations.

**Methods:**

Paraffin blocks of tissue samples from 150 patients who underwent colon surgery between 2018 and 2020 were prepared by the Pathology Department of Imam Hossein Hospital (Tehran, Iran). After DNA and RNA isolation, gene expression of miR-31 and miR-373 was determined using probe-based quantitative real-time polymerase chain reaction (qRT-PCR). Mutations of KRAS were surveyed using conventional PCR and agarose gel electrophoresis.

**Results:**

The mean age of the patients was 57.2 ± 13.4 years. KRAS codon 12 and 13 mutations were positive in 31 (20.6%) and 22 (14.6%) cases, respectively. The results showed that KRAS common mutations occurred in 32.6% of Iranian CRC patients. The expression levels of miR-31 and miR-373 increased in CRC patients with KRAS mutations in comparison with patients without these mutations.

**Conclusion:**

Considering the role of miR-31 and miR-373 in CRC tumor progression, it seems that the CRC patients bearing KRAS mutations have a poorer prognosis respective to patients without KRAS mutations.

## Introduction

Colorectal cancer (CRC) is thought to be closely related to lifestyle modifiers and age. The role of underlying genetic determinants has been proposed in some studies. Among the hereditary genetic abnormalities linked with CRC is familial adenomatous polyposis, which often emerges as a benign polyp but might gradually transform into a cancerous lesion [1, 2]. The CRC might be diagnosed by colonoscopy and pathologic examination. In addition, imaging studies might assist in distinguishing other organ involvement. Screening is effective in the prevention and reduction of mortality from CRC and is recommended for average-risk individuals with no family history of CRC initiated since 50 years of age. If there are small polyps in colonoscopy, they might be resected. Furthermore, biopsies are conducted for large polyps or tumors to check if the lesion is malignant [3, 4].

In most cases of CRC, an adenoma is initially detected, which subsequently transforms into carcinoma. Cancer progression can be effectively thwarted if these precancerous lesions are timely detected and resected, which requires CRC screening and diagnosis at early stages [5]. In 70% of sporadic cases of CRC, the lesions have been noted to develop from adenomatous polyps. In 25-30% of CRCs, sessile serrated lesions (SSLs) are the origin of cancer through the SSL-to-carcinoma pathway [6].

Screening for CRC is required for the diagnosis of this cancer at early stages and the initial detection and then removal of adenomas and SSLs. There are various screening tools available to detect adenomatous polyps, from computed tomography colonography and colonoscopy to sigmoidoscopy. In addition, stool-based testing (e.g., occult blood test) can be used to early detect cancerous changes. Colonoscopy is the preferred method to identify SSLs [7]. Studies are also seeking to find noninvasive and inexpensive molecular methods for CRC screening. Among these, recent research on CRC has established a link between the patient’s clinical and pathological conditions and several molecular and genetic markers [8, 9].

Recent research has shown that different clinical, histological, and molecular parameters can be valuable prognostic markers for CRC, which can also be used for the diagnosis and treatment of this cancer. The clinical value of these factors might be variable. For example, well-known histopathological markers are used to classify malignancies into distinct subtypes. On the other hand, the biological characteristics of tumors can be used to predict disease progression and choose appropriate therapeutic approaches. Recent studies have shown that molecular biomarkers can be promising tools for the better management of cancers [10, 11]. Among these molecular markers are small non-coding ribonucleic acids (RNAs). Previous studies have shown that these RNAs are aberrantly expressed in numerous malignant human tumors, including the prostate, colon, breast, bladder, liver, and brain tumors [12, 13]. The miR-31 is involved in the migration and invasion in breast and colorectal cancers [14, 15]. In CRC, miR-31 activates the RAS signaling pathway by inhibiting the RAS p21 GTPase activating protein 1. This property confers cancer cell growth and stimulates tumorigenesis. High expression of miR-31 is correlated with advanced disease and the worst clinical outcome in metastatic CRC [16]

Humans’ RAS genes (i.e., Kirsten rat sarcoma virus [KRAS], NRAS, and HRAS) are the most commonly mutated oncogenes in cancers, identified in 90% of pancreatic cancers, 35% of lung cancers, and 45% of CRCs [17]. The KRAS gene encodes KRAS, a protein acting in the downstream signaling pathways originated from the epidermal growth factor receptor (EGFR) [18]. Upon the interaction of the mentioned receptor with its ligand, the PI3K/AKT/MTOR signaling route is activated, inducing cellular proliferation [19].

The basis of the molecular pathogenesis of CRC is not well known and more molecular tests are needed to investigate the genes related to this disease. Also, if more detailed molecular pathways are discovered, more molecular treatments could be evaluated. Today, the treatment approaches are going to target malignant cells via genetic pathways to protect normal cells from the adverse effects of drugs. Therefore future studies should be focused on molecular tests.

The present study investigated two molecular markers (i.e., miR-31 and miR-373) involved in the pathogenesis of CRC and their association with demographic characteristics of patients, disease status, and histopathological features. In addition, the prognostic value of these molecular markers was investigated in CRC patients with or without common KRAS mutations.

## Methods

### Study design and patients

The paraffin blocks of tissue samples from 150 patients undergoing surgery on colon within 2018-2020 were prepared by the Pathology Department of Imam Hossein hospital in Tehran, Iran. The results of histopathological studies confirmed CRC diagnosis in these patients. The primary data were extracted from pathology report sheets, including demographic information, tumor type and location, and tumor differentiation status. Moreover, the information, including a history of smoking, family history of cancer, and preoperative treatments, were extracted from patients’ medical files.

### Molecular marker detection

The isolation of deoxyribonucleic acid (DNA) and RNA were performed using commercialized kits (Exgene FFPE Tissue DNA, and Hybrid-R™ miRNA kit, GeneAll, South Korea). Quantitative Reverse Transcription Kit (Qiagen, Germany) was used to synthesize complementary DNA from the extracted RNA. Specific primers and probes for the target genes were designed using Gene Runner software (version 3.05) and miRprimer software (version 2.0).

The gene expression of miR-31 and miR-373 was determined using probe-based reverse transcription-quantitative polymerase chain reaction (RT-qPCR). Primer sequences were 5′-CAGCTATGCCAGCATCTTGCCT-3′ for miR-31, 5′-GTCGTATCCAGTGCAGGGTCCGAGGT-3ˋ for miR-373, and 5′-CGAATTTGCGTGTCATCCT-3′ for U6 as control. The KRAS mutations were surveyed using conventional polymerase chain reaction (PCR) and agarose gel electrophoresis. Table 1 shows the primers used to detect KRAS common mutations.

**Table 1:** Primer sequences for detecting KRAS mutations

Mutation	Primer	Primer sequence (5′-3′)
KRAS Codon 12	Forward	GTTGTCGTAGTTGGAGCTGTTG
	Reverse	GGCACTCTTGCCTAC GCCAACAGC
KRAS Codon 13	Forward	GGTAGTTGGAGCTGGTGACGTAGGCA
	Reverse	GGCACTCTTGCCTAC GTCACCAGCT

### Data analysis

The expression of the target genes in the tumor tissues was determined by the calculation of the threshold cycles (C_T_) of the target and housekeeping (ctU6) genes. Then, relative gene expression was calculated using the standard formula. According to the default definition of the software system (version 5.0), a value < 50% difference was considered a nonsignificant change. The reduction or elevation of C_T_ was considered either the up-regulation or down-regulation of the target genes, respectively. Finally, significant differences in the expression of the target genes between the two groups were determined by appropriate statistical tests.

## Results

The present study investigated 150 patients with CRC. The majority of the patients (62%) were male, and the mean age of the patients was 57.2±13.4 years (age range: 27–88 years). Moreover, 34 patients (22.6%) had a family history of cancer; nevertheless, 16 patients (10.6%) had a family history of gastrointestinal cancers. Table 2 shows the patients’ demographic data in more detail.

**Table 2:** Patients’ demographic data

Variables	Result
Gender, male, *n* (%)	93 (62%)
Age (years)	57.2 ±13.4
BMI (kg/m^2^)	24.48 ± 5.60
Time between CRC diagnosis and surgery in patients (months)	8.2 ± 4.80
Smoking	61 (40.6%)
Family history of cancer	34 (22.6%)
Family history of gastrointestinal cancers	16 (10.6 %)

Tumor differentiation is categorized into three grades, including well-differentiated, moderately-differentiated, and poorly-differentiated. The results showed that the frequencies of well-differentiated, moderately-differentiated, and poorly-differentiated tumors were 49 (32.6%), 67 (44.6%), and 34 (22.8%), respectively. Table 3 shows other clinicopathological results.

**Table 3:** Clinicopathological features in patients with colorectal cancer

Subject		Result
Lymph node involvement (*n*/%)		12 (8%)
Distance metastasis (*n*/%)		6 (4%)
Vascular invasion (*n*/%)		5 (3%)
Tumor Diameter, mean ± SD (mm)		38.6 ±8.62
Localization of the tumor (*n*/%)	Proximal colon	80 (53.3%)
	Distal colon	70 (46.7%)

### Results of KRAS mutation analysis

Common mutations (two mutations) in the codons of 12 and 13 of the KRAS gene were investigated using conventional PCR and gel electrophoresis. The results are shown in Table 4.

**Table 4:** The results of KRAS mutation analysis

Mutations	Results	N (%)
KRAS codon 12 mutation	Positive	31 (20.6%)
	Negative	119 (79.4%)
KRAS codon 13 mutation	Positive	22 (14.6%)
	Negative	128 (85.4%)

### Gene expression data

Based on the results of the KRAS mutation analysis, the patients were divided into two groups, KRAS positive (*n*=49) and KRAS negative (*n*=110). Four patients showed both codon 12 and 13 mutations. The gene expressions of the target microRNAs (miRNAs) were analyzed in these two groups (Table 5).

**Table 5:** The relative gene expressions of miR-373 and miR-31 in colorectal cancer patients with or without KRAS mutations

Genes	Mean fold change (KRAS+/KRAS−)	***P*** value*
miR-37**3**	2.5	<0.001
miR-**31**	6.1	<0.001

*P value <0.05 was considered statistically significant

**Fig. 1:**
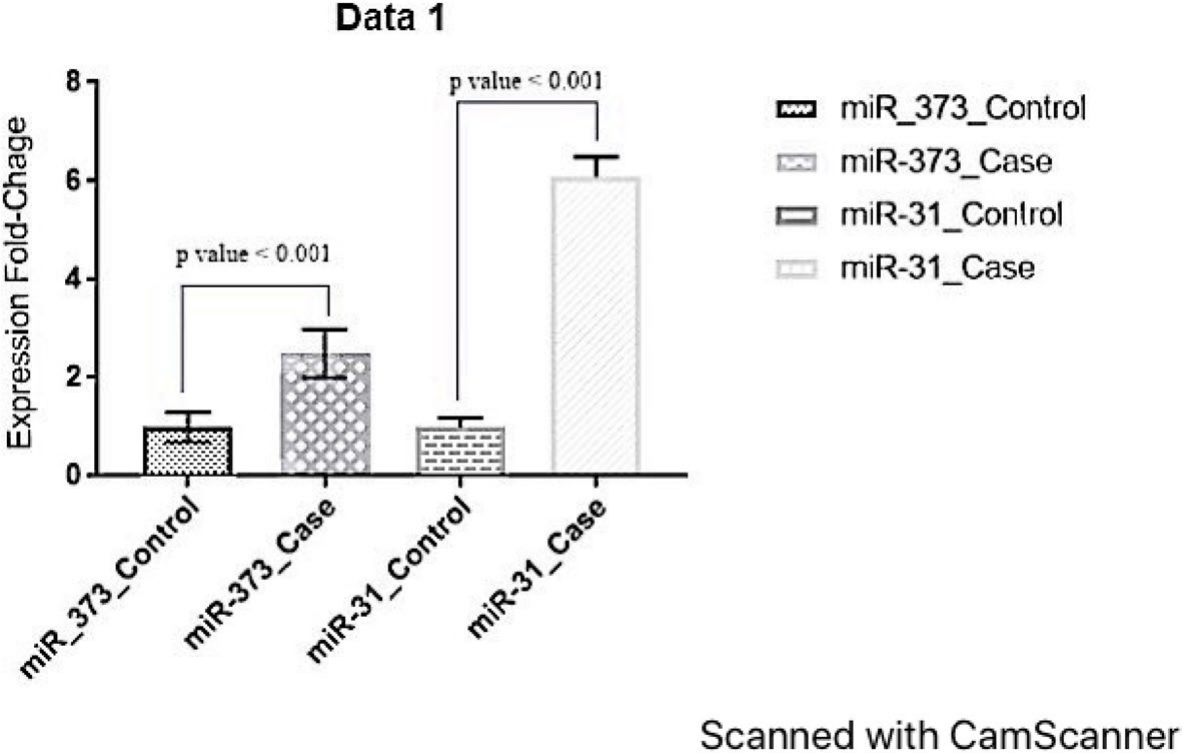
Expression fold-changes of miR-373 and miR-31

The results showed that the expression of miR-373 was significantly increased in KRAS-positive patients (with a mean fold-change of 2.5, *P*<0.001). Additionally, the expression of miR-31 was significantly increased in KRAS-positive patients (with a mean fold-change of 6.1, *P*<0.001), which is shown in Fig. 1.

The association of several clinicopathological factors with the expression levels of miR-31 and miR-373 was investigated in CRC patients. The Mann-Whitney *U* test was used for comparisons between two groups, and the Kruskal-Wallis test was utilized for three or more groups (Table 6). The results showed that the odds ratios of miR-31 and miR-373 were suggestive of tumor differentiation.

**Table 6:** Comparison of miR-31 and miR-373 expressions based on several clinicopathological factors in CRC patients

Clinicopathological factors		miR-31	miR-373
Lymph node involvement	Yes	0.88 (0.11–1.78)	0.81 (0.01–1.42)
	No	0.93 (0.14–1.86)	0.83 (0.11–1.76)
		*P* value=0.81	*P* value=0.66
Distance metastasis	Yes	1.02 (0.06–2.01)	1.00 (0.1–2.21)
	No	0.98 (0.51–1.68)	0.81 (0.3–1.55)
		*P* value=0.74	*P* value=0.54
Tumor diameter	<40 mm	1.12 (0.81–2.22)	1.02 (0.3–1.99)
	>40 mm	1.02 (0.75–1.99)	0.99 (0.42–1.78)
		*P* value=0.88	*P* value=0.66
Tumor location	Proximal colon	0.97 (0.41–1.66)	1.31 (0.31–2.43)
	Distal colon	0.99 (0.45–1.76)	1.22 (0.65–2.00)
		*P* value=0.5	*P* value=0.88
Tumor differentiation	Well	3.1 (1.5–4.43)	8.1 (2.31–10.22)
	Moderate	0.91 (0.81–1.87)	1.44 (0.88–2.76)
	Poor	0.5 (0.1–1.1)	0.88 (0.11–1.99)
		*P* value=0.001	*P* value=0.001

## Discussion

The CRC is the third cause of mortality among patients with cancer of both genders. Appropriate screening has been shown to be effective in the reduction of CRC morbidity and mortality [19]. The expression of nearly one-third of human coding genes is somehow regulated after transcription via various miRNAs. These small RNAs are frequently observed to have aberrant expressions in various tumors; nevertheless, their exact role in cancer pathogenesis is yet to be divulged.

This study investigated miR-31 and miR-373 genes’ expressions in 150 patients with CRC. Furthermore, this study compared the expression of these two miRNAs between CRC patients with or without two common mutations in the KRAS gene. The KRAS gene encodes KRAS, a protein acting in the downstream signaling pathways originated from the epidermal growth factor receptor [18]. Upon the interaction of the aforementioned receptor with its ligand, the PI3K/AKT/mTOR signaling route is activated, inducing cellular proliferation. Point mutations in codons 12 and 13 of the KRAS gene comprise about 90% of the mutations identified in CRC patients. The mutations of Gly→Val (G12V) in codon 12 and Gly→Asp (G13D) in codon 13 are the most commonly detected mutations in the KRAS gene [20, 21].

The results of the present study showed that the frequencies of codon 12 and 13 mutations were 20.6% and 14.6%, respectively. Four of the cases revealed mutations in both codon 12 and codon 13. Collectively, 49 patients (32.6%) were detected with KRAS mutations, which showed a similar rate as reported in previous studies in Iran. A study performed by Koochak et al. demonstrated KRAS mutations in 33.6% of cases. However, the number of patients enrolled in the aforementioned study was far greater than in the present study (1000 vs. 150). Similarly, 32% of patients revealed KRAS mutations in a study by Omidifar et al. [22].

The current study also showed that CRC patients harboring KRAS mutations had significantly upregulated expressions of both miR-31 and miR-373, compared to those who were tested negative for KRAS mutations. In line with the aforementioned results, Xin et al. comparing miR-31 gene expression between normal mucosal tissues and CRC tumors showed the significant upregulation of this miRNA [23]. Yan Song et al., in their study, demonstrated the upregulation of miR-31 in ulcerative colitis and noted that the suppression of special AT-rich DNA-binding protein 2 (SATB2) can be a possible route through which this miRNA might promote tumorigenesis [24]. In another study, Lundberg et al. investigated microRNA expression in KRAS and B-Raf mutated CRC patients. Their results showed that RAF-mutated tumors were found to express significantly higher levels of miR-31 as well as significantly lower levels of miR-373, compared to wild-type tumors, so they suggested that KRAS- and BRAF-mutated CRCs may have different miRNA signatures compared to CRC tumors wild-type in KRAS and BRAF [25]

The present study’s findings are in line with those reported by the aforementioned studies.

It is postulated that miR-372 and miR-373 might block the differentiation of CRC cells, enhancing their stemness. It is not yet well characterized how miR-31 promotes its mechanisms involved in cancer progression. Findings on the role of this miRNA as a tumor suppressor or oncogene are not conclusive. Some researchers have declared a tumor-suppressive role for miR-31 by targeting the PI3K/AKT pathway and Rho protein. This miRNA has been proposed to promote an oncogenic role by inducing several signaling pathways, such as RAS/MARK/ERK1/2. The present study’s observation about the correlation between tumor differentiation and miR-31 expression can be justified in part by the awareness of the fact that the KRAS protein acts in the PI3K/AKT/mTOR pathway.

One limitation in the current study was the use of formalin-fixed paraffin-embedded samples and that RNA stability might be compromised in these samples, especially during long storage periods. However, due to the high stability of miRNAs, it has been confirmed that it is possible to determine the expression of miRNAs in these samples using standardized RT-qPCR.

## Conclusion

The results showed that KRAS common mutations occurred in 32.6% of the studied CRC patients. The expression levels of miR-31 and miR-373 increased in CRC patients with KRAS mutations in comparison to patients without these mutations. Considering the role of miR-31 and miR-373 in CRC tumor progression, it seems that the CRC patients bearing KRAS mutations have a poorer prognosis in comparison to patients without KRAS mutations.

## Data Availability

The datasets generated and/or analyzed during the current study are not publicly available, but are available from the corresponding author on reasonable request.

## Abbreviations

CRC: Colorectal cancers
QRT-PCR: Quantitative real-time polymerase chain reaction
SSLs: Sessile serrated lesions
CT colonography: Computed tomography colonography
DNA: Deoxyribonucleic acid
RNA: Ribonucleic acid
PCR: Polymerase chain reaction
